# Supplementary figures and images for: Inflammatory Response in Caco-2 Cells Stimulated with Anisakis Messengers of Pathogenicity
Source: Pathogens. 2022 Oct 20;11(10):1214. doi: 10.3390/pathogens11101214 (PMC9611079; doi:10.3390/pathogens11101214)

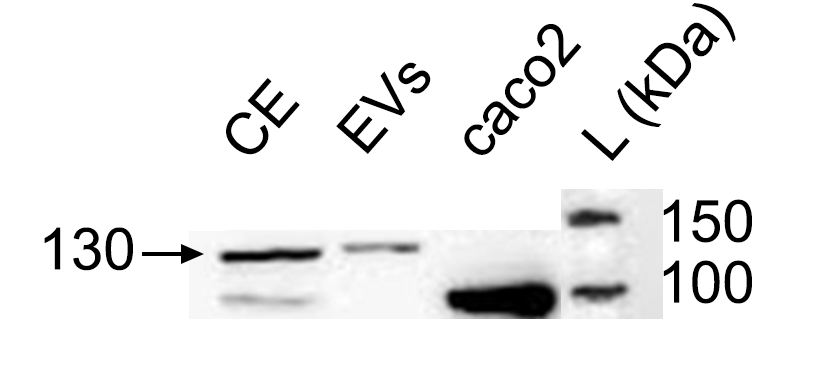

Supplement: Supplementary file 1 [file pathogens-11-01214-s001.zip › Supplementary/Figure S1]
